# Impact of a deep-learning image reconstruction algorithm on image quality and detection of solid lung lesions

**DOI:** 10.1016/j.redii.2025.100062

**Published:** 2025-05-27

**Authors:** Joël Greffier, Maxime Pastor, Quentin Durand, Renaud Sales, Chris Serrand, Jean-Paul Beregi, Djamel Dabli, Julien Frandon

**Affiliations:** a Imagine UR UM 103, université de Montpellier, Department of Medical Imaging, centre hospitalier universitaire de Nîmes, Nîmes, France; b Department of Biostatistics, Clinical Epidemiology, Public Health, and Innovation in Methodology (Bespim), centre hospitalier universitaire de Nîmes, 30029 Nîmes, France

**Keywords:** Artificial intelligence, Deep learning image reconstruction, Multidetector computed tomography, Image enhancement, Chest tumor

## Abstract

**Purpose:**

To compare the impact of a deep-learning image reconstruction algorithm (Precise Image) with an iterative reconstruction algorithm on image quality and detection of solid lung lesions in chest CT images.

**Methods:**

All consecutive patients with at least one solid lung lesion diagnosed between December 2021 and February 2022 were retrospectively included. Images were reconstructed using Level 4 of the iterative reconstruction algorithm (i4) and the Standard/Smooth/Smoother levels of the deep-learning image reconstruction algorithm. Mean attenuation and standard deviation were measured by placing regions of interest in fat, muscle, trachea and solid lung lesions. The contrast-to-noise ratio between the lesion and the trachea was computed. Two radiologists assessed image noise and image smoothness, overall image quality and confidence diagnostic level using Likert scales. One radiologist also measured the large axis of the largest lesion. Statistical analyses was performed to compare outcomes obtained with the different algorithms.

**Results:**

Thirty patients with a mean age of 70.0 ± 9.0 years (17 men) were included. The mean CTDI_vol_ was 6.3 ± 2.1 mGy. For all tissues, the contrast-to-noise ratio was similar for i4 and Standard level (*p* > 0.05) but increased significantly with other deep-learning image reconstruction levels compared to i4 (*p* < 0.05) and increased significantly from Standard to Smoother. Radiologists rated the image noise with a similar score between i4 and Standard level but decreased significantly between i4 and other deep-learning image reconstruction levels (*p* < 0.05) and from Standard to Smoother levels (*p* < 0.01). Overall image quality score were highest for the Smooth and Smoother levels.

**Conclusion:**

Smooth and Smoother levels may now be used in clinical practice for chest CT acquisitions in solid lung lesion follow-up.

## 1 Introduction

Patients with a history of cancer are exposed to repeated CT scans during their diagnosis and follow-up, with cumulative doses sometimes exceeding 100 mSv [1]. Low-dose chest and/or abdomen-pelvis CT scans are established as a promising means of early detection and diagnosis of cancerous lesions, while significantly reducing radiation exposure [[2], [3], [4], [5], [6], [7]–8]. The emergence of these low-dose acquisitions has been made possible by the use of iterative reconstruction algorithms. However, the properties of these algorithms can lead to image smoothing, which can interfere with the radiologist's interpretation [9].

In recent years, with the development of artificial intelligence solutions in healthcare, new algorithms based on deep-learning have been developed to reconstruct CT images. Compared with iterative reconstruction algorithms, they reduce noise and artefacts in images whist preserving spatial resolution. These deep-learning image reconstruction algorithms are grouped into two main categories (i.e., direct and indirect) and are released by independent software companies and CT vendors [10]. Two algorithms proposed by GE Healthcare and Canon Medical Systems use a deep-neural network trained with high-quality datasets. One uses datasets derived from patient images reconstructed with a model-based iterative reconstruction algorithm and the other uses datasets derived from patient and phantom images reconstructed with filtered-back projection [11][12,13]. In 2021, another CT vendor (Philips Healthcare) developed a new deep-learning image reconstruction algorithm. As explained in previous studies, this algorithm does not use a deep-neural network but a convolutional neural network trained to reproduce the image appearance of routine-dose filtered back-projection images from the raw data of low-dose CT scans [[14], [15], [16], [17]–18]. As described by Koetzier et al., the performance of each algorithm depends on the quality of the datasets used for its development [10].

Numerous studies have demonstrated the impact of deep-learning image reconstruction algorithms on improving image quality and the potential for dose reduction, particularly for chest CT examinations [[19], [20], [21], [22], [23], [24], [25], [26], [27], [28]–29]. However, as far as we know, there have been no studies to evaluate the impact of the newest algorithm on the image quality of chest CT and the follow-up of solid lung lesions in oncology. Preliminary studies have been made on phantoms with this algorithm and one preliminary study on patients validated the quality of images obtained for low-dose abdomen-pelvis CT acquisitions for the detection and follow-up of liver metastases [14], [15], [16], [17][30].

The aim of this study was to compare of the latest deep-learning image reconstruction algorithm with that of an iterative reconstruction algorithm on the impact on image quality and detection of solid lung lesions. To this end, parenchymal chest images from low-dose chest-abdomen-pelvis CT acquisition were studied.

## 2 Methods

### 2.1 *Patients*

We enrolled all consecutive eligible adult patients with at least one chest tumor (solid lung lesion) diagnosed by a previous chest-abdomen-pelvis CT scan, undergoing follow-up CT scans within the inclusion period December 2021 - February 2022. These patients underwent the regular CT protocol at our institution with the conventional acquisition and reconstruction parameters, but the raw data was also retrospectively reconstructed with the new deep-learning image reconstruction algorithm. Patients under 18 years of age and patients who did not meet the inclusion criteria were excluded from the study.

Our institutional review board (21.11.03) had given full approval for this retrospective monocentric study. Participants and/or their legal guardians were systematically informed that details were being collected for an anonymous retrospective study and could refuse to participate in it at any time.

### 2.2 *CT protocol*

An Incisive CT Premium (Philips Healthcare) CT system was used to perform chest-abdomen-pelvis acquisitions. A tube voltage of 100 kVp (120 kVp for overweight patients), a pitch factor of 1.2, a rotation time of 0.35 s/rot and a physical beam collimation of 64 × 0.625 mm were used as acquisition parameters. The automatic tube current modulation system was used with the Dose Right Index set at 15 to be close to a volume CT dose index (CTDI_vol_) of 6 mGy [16]. This dose level corresponds to the low-dose level in France (25th percentile of national diagnostic reference level data distribution).

A standard power injector was used to inject low osmolar iodinated contrast media at an injection rate of 3–5 mL/s and the total volume injected was adjusted according to the patient’s body weight (2 mL/kg). Seventy seconds after starting the injection, the chest-abdomen-pelvis acquisitions were made at the portal phase.

In this study, only parenchymal chest images were evaluated. Level 4 of the iDose^4^ (iterative reconstruction) algorithm (i4) and the Standard, Smooth and Smoother levels of the Precise Image (deep-learning image reconstruction) algorithm were used to reconstruct the raw data [16]. The “YA” reconstruction kernel was used for i4 and the “Lung” reconstruction kernel was used for the deep-learning image reconstruction algorithm. A slice thickness of 1 mm (0.5 mm overlapped) was used with i4 and deep-learning image reconstruction.

### 2.3 *Dosimetry evaluation*

The CTDI_vol_, the dose length product, the size-specific dose estimate and the average scan size calculated by the CT system for the chest-abdomen-pelvis CT acquisition were retrieved from each patient’s report at the end of the acquisitions.

### 2.4 *Chest tumor evaluation*

The solid tumors (solid lung lesions) were classified according to the number and size of tumors. For each reconstruction type, a senior radiologist with 10-year experience (R1; JF) also measured the large axis of the largest chest tumor on axial images. Last, the largest chest tumors were classified into on of two groups according to the classification proposed by Fitton et al. [31]. Group I corresponds to the largest chest tumor with any of the following features: surrounded by lung or visceral pleura (no atelectasis), without venous invasion, or extending to the chest wall or the mediastinum over less than one-quarter of its surface. Group II corresponds to the largest chest tumor which has invaded any of the following: the hilar region, heart, large vessels, pericardium, mediastinum over more than a quarter of its surface, or association atelectasis (Table 1).

**Table 1:** Results of a study comparing the impact of a deep-learning image reconstruction algorithm with an iterative reconstruction algorithm on image quality and detection of solid lung lesions in chest CT images: patient characteristics.

	Values
Age (years)	70.2 ± 8.6 [51 - 86]
Sex (women/men)	11 (36.7 %)/19 (63.3 %)
Patients receiving chemotherapy	22 (73.3 %)
Primary neoplasm	
Colorectal cancer	14 (46.7 %)
Lung cancer	7 (26.7 %)
Pancreatic cancer	2 (6.7 %)
Uterine cancer	2 (6.7 %)
Renal cancer	2 (6.7 %)
Breast cancer	1 (3.3 %)
Tongue cancer	1 (3.3 %)
Number of lung lesions	
1 to 3	20 (66.7 %)
3 to 10	2 (6.7 %)
> 10	8 (26.7 %)
Size of lung lesions	
< 1 cm	3 (10.0 %)
1 to 3 cm	18 (60.0 %)
3 to 10 cm	9 (30.0 %)
Anatomical location of the larges chest tumor	
Group I – Lung/tumor	13 (43.3 %)
Group I – Chest wall/tumor	10 (33.3 %)
Group I – Mediastinum/tumor	2 (6.7 %)
Group II – Mediastinum, hilum/tumor	3 (10.0 %)
Group II – Atelectasis/tumor	2 (6.7 %)
Volume of iodine injected (mL)	77.9 ± 12.3 [50 - 120]

Values are expressed as means ± standard deviations [min - max] or the number of patients (percentage).

### 2.5 *Objective image quality assessment*

The quality of each image was objectively assessed by a junior radiologist with 5 years of experience (R2; QD) on the manufacturer’s workstation (IntelliSpace Portal, Philips Healthcare). Four regions of interest were placed in the subscapular muscle, trachea, largest chest tumor and axillary fat. The mean (N_CT_) and standard deviations (image noise) of pixel values were computed, and the contrast-to-noise ratio (CNR) was calculated as follows:

$$CNR=\frac{\left|N_{CT,\;tumor}-N_{CT,\;trachea}\right|}{Noise_{trachea}}$$

As the structure and contents of the trachea are homogeneous from one patient to another (unlike the lung parenchyma), the N_CT_ and image noise measured in the region of interest placed within it were used to calculate the contrast-to-noise [32].

### 2.6 *Subjective image quality assessment*

All the chest images were read by two radiologists (R1 and R2), blinded to the reconstruction type (algorithm and level), who assessed the image noise on a standard 5-point scale [15] in which 1 = unacceptable, 2 = suboptimal, 3 = acceptable, 4 = above average, and 5 = excellent. They also assessed image smoothing (including image texture and/or the presence of artefacts) using the following five-point scale in which 1 = no image smoothing, 2 = little image smoothing, 3 = moderate image smoothing, 4 = considerable image smoothing, and 5 = complete image smoothing. A previously published scale [14,15] was used to rate the overall image quality (1 = not evaluable, 2 = interpretable despite moderate artifacts or noise, 3 = fully interpretable with mild artefacts or noise, 4 = no artifacts or noise). Diagnostic image quality including lesion visibility (1 = unacceptable; 2 = suboptimal; 3 = acceptable; 4 = above average; 5 = excellent) was also assessed.

### 2.7 *Statistical analyses*

An in-house biostatistician (C.S) performed the statistical analyses using SAS v9.4 software. Normality was explored graphically and via the Shapiro-Wilk test for all quantitative data [33] and these were expressed as means ± standard deviations (SD) and medians with first and third quartiles as required.

The two-tailed Wilcoxon signed-rank test was used to determine differences in N_CT_, image noise, CNR and the large axis of the largest chest tumor values and ordinal variables between i4 and all DLR levels. A *p*-value of <0.05 was considered significant.

Gwet's AC2 agreement coefficient was estimated with its 95 % confidence interval to evaluate agreement between the two radiologists [34,35]. An estimate less than 0.4 was considered as “poor agreement”, 0.4 to 0.6 was considered as “fair agreement”, 0.6 to 0.8 as “good agreement” and greater than 0.8 as “excellent agreement” [35]. If both readers gave an identical score for all images, no concordance coefficient could be calculated.

## 3 Results

### 3.1 *Patients*

Thirty patients with known chest tumors (solid lung lesions) were enrolled (Table 1).

The primary neoplasm was colorectal cancer (*n* = 14, 46.7 %), followed by lung cancer (*n* = 7, 26.7 %), pancreatic cancer (*n* = 2, 6.7 %), uterine cancer (*n* = 2, 6.7 %); renal cancer (*n* = 2, 6.7 %) breast cancer (*n* = 1, 3.3 %) and tongue cancer (*n* = 1, 3.3 %) (Table 1). Of the twenty-two patients who received chemotherapy, CT scans were performed before treatment for two patients (4.5 %), during treatment for one patient (9.1 %) and after treatment for 19 patients (86.4 %).

The largest chest tumor was classified as Group I for 83.0 % of patients and as Group II for 17.0 % (Table 1). For Group I, the largest chest tumor was surrounded by lung or visceral pleura (no atelectasis), without venous invasion for 13 patients and extending to the chest wall for 10 patients.

### 3.2 *Dosimetry*

The mean CTDI_vol_ for chest-abdomen-pelvis CT acquisition (6.3 ± 2.1 mGy) and the dose length product (444.3 ± 176.3 mGy.cm). The mean average scan size was 29.9 ± 2.9 cm and the mean size-specific dose estimate (SSDE) was 7.5 ± 1.7 mGy.

The mean tube voltage used was 106.7 ± 9.6 kVp although a tube voltage of 120 kVp was used for 10 patients with the highest average scan size (28.3 ± 1.9 cm at 100 kVp and 32.9 ± 1.9 cm at 120 kVp).

### 3.3 *Objective image quality assessment*

For trachea, the mean CT attenuation was higher with i4 than with all deep-learning image reconstruction levels (*p* < 0.05) and decreased significantly from Standard to Smoother levels (*p* < 0.05; Table 2, Table 3). For other tissues, similar mean CT attenuation values were found between i4 and DLR and between all DLR levels (*p* > 0.05).

**Table 2:** Results of a study comparing the impact of a deep-learning image reconstruction algorithm with an iterative reconstruction algorithm on image quality and detection of solid lung lesions in chest CT images: image quality assessment.

	iDose^4^ level 4	Standard	Smooth	Smoother
Objective image quality assessment				
Mean attenuation (HU)				
Fat	−109 (−115; −105)	−109 (−114; −105)	−110 (−115; −105)	−112 (−118; −105)
Muscle	52 (44; 58)	50 (44; 57)	55 (44; 60)	53 (46; 59)
Trachea	−979 (−992; −973)	−982 (−996; −975)	−985 (−999; −974)	−989 (−1000; −982)
Tumor	58 (48; 69)	60 (47; 74)	62 (53; 76)	64 (46; 77)
Image noise (HU)				
Fat	38.9 (36.4; 46.0)	38.2 (34.2; 42.2)	27.1 (23.7; 30.4)	16.8 (14.1; 19.3)
Muscle	52.0 (47.2; 61.6)	52.1 (43.1; 59.0)	37.4 (30.1; 45.4)	19.6 (17.1; 23.4)
Trachea	39.5 (34.3; 45.7)	40.4 (28.1; 47.4)	29.4 (18.6; 37.2)	20.6 (13.3; 29.4)
Tumor	50.1 (43.7; 57.0)	52.2 (44.4; 61.7)	42.1 (37.2; 46.0)	26.5 (19.5; 33.4)
Contrast-to-noise ratio, tumor	26.2 (22.7; 30.3)	25.6 (21.6; 36)	35.7 (28; 57.1)	50.9 (36.5; 81.9)
Large axis of the largest chest tumor	20.6 (16.8; 35.0)	21.9 (16.6; 34.7)	22.0 (16.6; 34.2)	21.8 (16.5; 34.6)
Subjective image quality assessment				
Image noise				
Score	3.0 (3.0; 3.0)	3.0 (3.0; 3.0)	4.0 (4.0; 4.0)	4.5 (4.5; 4.5)
Gwet AC2 [95 % CI]	0.97 [0.90; 1.00]	–	0.89 [0.76; 1.00]	–
Image smoothing				
Score	1.0 (1.0; 1.0)	2.0 (2.0; 2.0)	3.5 (3.5; 3.5)	4.5 (4.5; 4.5)
Gwet AC2 [95 % CI]	–	–	0.93 [0.83; 1.00]	–
Overall image quality				
Score	2.5 (2.5; 2.8)	3.5 (3.5; 3.5)	3.8 (3.8; 3.8)	4.0 (4.0; 4.0)
Gwet AC2 [95 % CI]	0.46 [0.26; 0.66]	–	0.95 [0.86; 1.00]	0.90 [0.82; 0.99]
Diagnostic image quality				
Score	3.5 (3.5; 3.5)	4.5 (4.5; 4.5)	4.5 (4.5; 4.5)	5.0 (5.0; 5.0)
Gwet AC2 [95 % CI]	0 [0; 0]	0.07 [0 ; 0.18]	0 [0; 0]	–

Quantitative and qualitative values are expressed as medians [1st quartile; 3rd quartile]. For the Gwet AC test, figures correspond to a 95 % confidence interval. If both readers gave an identical score for all images, no concordance coefficient could be calculated, and this is indicated as "-". CI: confidence interval; HU: Hounsfield units.

**Table 3:** Results of a study comparing the impact of a deep-learning image reconstruction algorithm with an iterative reconstruction algorithm on image quality and detection of solid lung lesions in chest CT images: *p*-values calculated for all variables between Level 4 of the iterative reconstruction algorithm and the three levels of the deep-learning image reconstruction algorithm.

Comparaison	i4 vs. Standard	i4 vs. Smooth	i4 vs. Smoother	Standard vs. Smooth	Standard vs. Smoother	Smooth vs. Smoother
N_CT_ fat	0.54	0.27	0.29	0.55	0.35	0.29
N_CT_ muscle	0.66	0.20	0.49	0.31	0.42	0.41
N_CT_ trachea	**0.02**	**<0.001**	**<0.0001**	**0.01**	**<0.001**	**0.01**
N_CT_ tumor	0.19	0.06	0.08	0.47	0.15	0.75
Noise fat	0.33	**<0.0001**	**<0.0001**	**<0.0001**	**<0.0001**	**<0.0001**
Noise muscle	0.74	**<0.0001**	**<0.0001**	**<0.0001**	**<0.0001**	**<0.0001**
Noise trachea	0.09	**<0.0001**	**<0.0001**	**<0.0001**	**<0.0001**	**<0.01**
Noise tumor	0.40	**<0.0001**	**<0.0001**	**<0.0001**	**<0.0001**	**<0.001**
CNR	0.20	**<0.0001**	**<0.0001**	**<0.0001**	**<0.0001**	**<0.0001**
Large axis of the largest chest tumor	0.37	0.59	0.74	0.58	0.41	0.85
Image noise	0.50	**<0.0001**	**<0.0001**	**<0.0001**	**<0.0001**	**<0.0001**
Image smoothing	**<0.0001**	**<0.0001**	**<0.0001**	**<0.0001**	**<0.0001**	**<0.0001**
Objective image quality	**<0.0001**	**<0.0001**	**<0.0001**	**<0.0001**	**<0.0001**	**0.02**
Diagnostic image quality	**<0.0001**	**<0.0001**	**<0.0001**	0.50	**<0.0001**	**<0.0001**

CNR: contrast-to-noise ratio; i4: level 4 of the iDose^4^ algorithm; N_CT_ corresponds to the mean attenuation. *P*-values lower than 0.05 were considered significant (bold).

For all tissues, image noise was similar between i4 and the Standard level (*p* > 0.05) but significantly higher with i4 than with Smooth (−29.0 ± 22.5 %) and Smoother (−50.1 ± −31.5 %) levels (*p* < 0.05). The image noise decreased significantly from Standard to Smoother levels (*p* < 0.05). For the CNR, the opposite pattern was found.

Similar values for the large axis of the largest chest tumor were found between i4 and deep-learning image reconstruction and between all deep-learning image reconstruction levels (*p* > 0.05).

### 3.4 *Subjective image quality assessment*

The two radiologists found that image noise was similar between i4 and the Standard level (*p* = 0.50) but decreased significantly from the Standard to Smoother level (*p* < 0.05) (Table 2, Table 3). For image smoothing, the score increased significantly between i4 and all deep-learning image reconstruction levels and from Standard to Smoother levels (*p* < 0.05). For these two criteria, there was excellent agreement between the two radiologists.

**Fig. 1:**
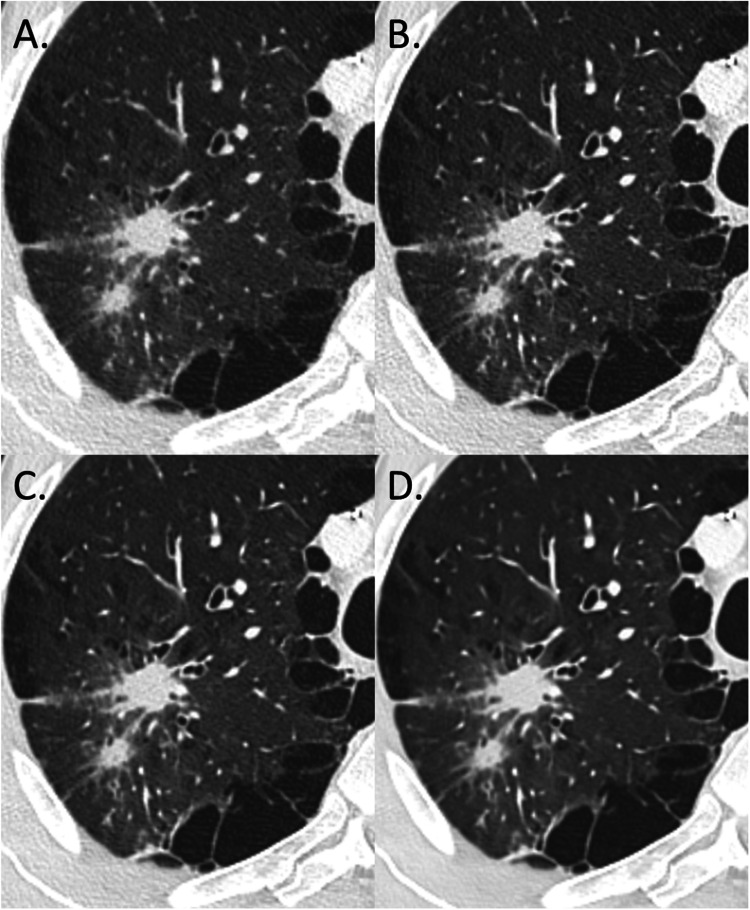
Results of a study comparing the impact of a deep-learning image reconstruction algorithm with an iterative reconstruction algorithm on image quality and detection of solid lung lesions in chest CT images: overall image quality of chest CT images (WL: −600 HU; WW: 1600 HU) of a man with spiculated mass in the right upper lobe (Group I – Lung/tumor) from primary lung cancer (62 years old; SSDE: 7.61 mGy; average scan size: 29.9 cm). (A) IR level 4; average overall image quality score: 3; (B) DLR, Standard; average overall image quality score: 3; (C) DLR, Smooth; average overall image quality score: 3.5; (D) DLR, Smoother; average overall image quality score: 4. DLR: deep-learning image reconstruction algorithm; HU: Hounsfield units; IR: iterative reconstruction; SSDE: size-specific dose estimate.

**Fig. 2:**
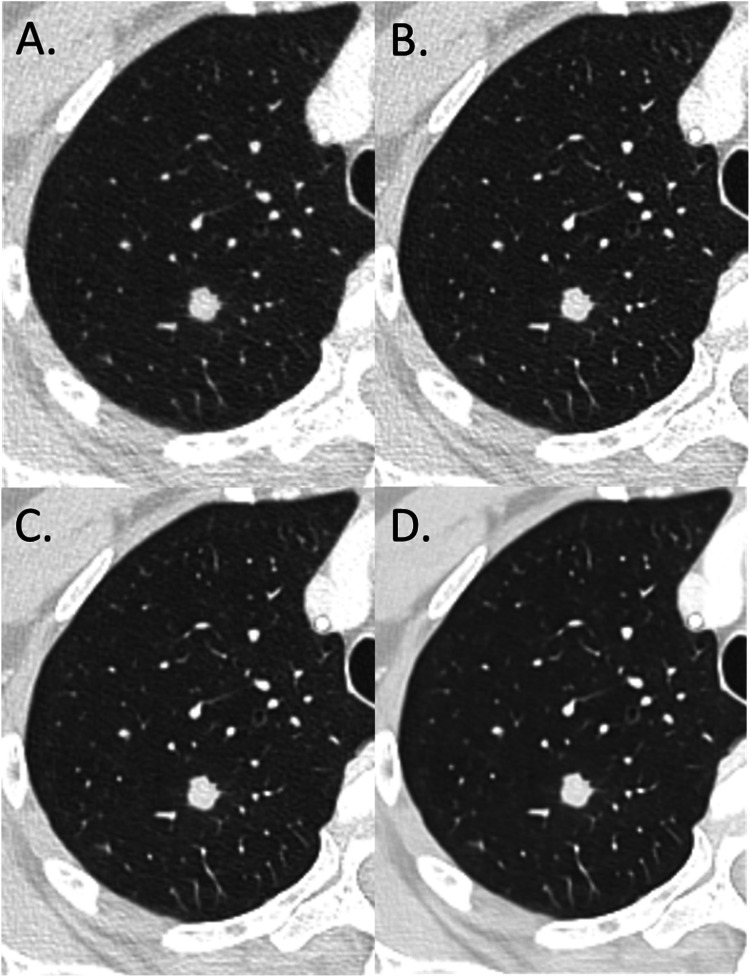
Results of a study comparing the impact of a deep-learning image reconstruction algorithm with an iterative reconstruction algorithm on image quality and detection of solid lung lesions in chest CT images: chest CT images (WL: −600 HU; WW: 1600 HU) of a woman with secondary pulmonary nodule in the right upper lobe (Group I – Lung/tumor) from primary colorectal cancer (60 years old; SSDE: 5.69 mGy; average scan size: 26.2 cm). (A) IR level 4; (B) DLR, Standard; (C) DLR, Smooth; (D) DLR, Smoother. DLR: deep-learning image reconstruction algorithm ; IR : iterative reconstruction algorithm; HU: Hounsfield units; SSDE: size-specific dose estimate.

**Fig. 3:**
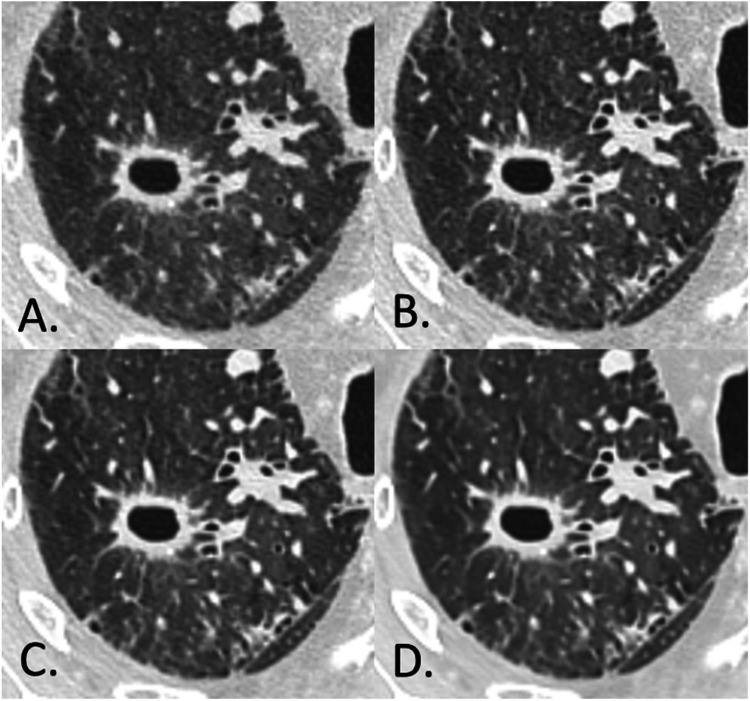
Results of a study comparing the impact of a deep-learning image reconstruction algorithm with an iterative reconstruction algorithm on image quality and detection of solid lung lesions in chest CT images: chest CT images (WL: −600 HU; WW: 1600 HU) of a woman with an excavated pulmonary nodule (Group I – Lung/tumor) fromprimary lung cancer (85 years old; SSDE: 8.07 mGy; average scan size: 30.8 cm). (A) IR level 4; (B) DLR, Standard; (C) DLR, Smooth; (D) DLR, Smoother. DLR: deep-learning image reconstruction algorithm ; IR : iterative reconstruction algorithm; HU: Hounsfield units; SSDE: size-specific dose estimate.

The overall image quality score increased significantly from i4 to the Standard level (*p* < 0.05) and from Standard to Smoother (*p* < 0.05) (Figs. 1, Fig. 2, Fig. 3). With i4, for both radiologists, and for all patients, the overall image quality was rated as “Interpretable despite moderate artefacts or noise”. The radiologists rated the overall image quality of the Standard and Smooth levels as “Fully interpretable with mild artefacts or noise” or as “No artefacts or noise”. For the Smoother level, they rated the overall image quality as “no artifacts or noise” for all patients. Agreement between the two radiologists was “excellent” for all deep-learning image reconstruction levels but “fair” for i4 with a mean score of 2.2 ± 0.3 for R1 and 3.0 ± 0.3 for R2.

The diagnostic image quality score increased significantly from i4 to the Standard level (*p* < 0.05) and from Standard to Smoother (*p* < 0.05), except from Standard to Smooth levels for the diagnostic image quality (Figs. 1, Fig. 2, Fig. 3). The radiologists rated the diagnostic image quality as “above average” or “excellent” for the three deep-learning image reconstruction levels and “acceptable” or “above average with i4. Agreement between the two radiologists was “excellent” the Smoother level and “fair” for i4 and other deep-learning image reconstruction levels.

## 4 Discussion

In the present study, we compared the quality of chest CT images obtained with the new deep-learning image reconstruction algorithm, developed by Philips Healthcare (Precise Image) and with an iterative reconstruction algorithm. Compared with the iDose^4^ level 4 usually used in clinical routine, the Smooth and Smoother levels of the new algorithm improved the image quality without altering lesion size.

The results of the objective image quality assessment showed that, for all tissues apart from the trachea, the mean CT attenuation values were similar between i4 and the three deep-learning image reconstruction levels. For the trachea, the mean CT attenuation values decreased between i4 and the deep-learning image reconstruction levels, as the deep-learning image reconstruction level increased. Regarding image noise outcomes, similar noise values were found for all tissues between i4 and the Standard level, and the noise decreased from Standard to Smoother. For the same dose level and reconstruction kernels (i.e. YA and Lung), similar outcomes were found in a preliminary phantom study between deep-learning image reconstruction levels [16]. However, in this study, noise magnitude was higher with the Standard level than with i4. The combined results of image noise and mean CT attenuation resulted in an increase in contrast-to-noise ratio between tumor and trachea from Standard to Smoother levels. The highest contrast-to-noise ratio values were found with the Smooth and Smoother levels compared to i4 and the Standard level. Similar contrast-to-noise ratio values were found between i4 and the Standard level. Similar results were found in the phantom study for the high-contrast lung lesion detectability index [16]. Finally, measurements of the large axis of the largest tumor taken by a radiologist showed that using the deep-learning image reconstruction algorithm versus i4 had no impact on lesion size. Similar values were found between i4 and deep-learning image reconstruction levels.

The results of the subjective image quality assessment confirmed the outcomes obtained on the objective image assessment for image noise. Both radiologists rated that the image noise was similar between i4 and Standard and decreased from Standard to Smoother levels. They also found that the image smoothing increased using deep-learning image reconstruction compared to i4 and as the deep-learning image reconstruction levels increased. Similar outcomes were found in the preliminary phantom study, where the image texture changed (noise power spectrum curves shift towards lower frequencies) from Standard to Smoother levels [16]. Finally, even though there was greater image smoothing, the decrease in image noise led to an increase in both radiologist’s overall image quality and diagnostic image quality scores as deep-learning image reconstruction levels increased. These scores were higher with the Smooth and Smoother levels than with i4 and the Standard level. For the latter two, the overall image quality score was higher with the Standard level than i4 but the agreement between the two radiologists was fair for i4 images. In fact, although their agreement was excellent for all the criteria evaluated and all types of images, one radiologist was stricter than the second regarding the overall image quality of i4 images. This difference in perception between the two radiologists was even more marked for the diagnostic image quality score. Although scores ranged from "acceptable" to "excellent" for all patients and all image types, agreement between the two radiologists was “fair” for i4, Standard and Smooth. These differences may be explained by the subjective nature of this type of assessment.

Based on the combined results of the objective and subjective analyses, the Smooth and Smoother levels offered the best results, and better results than the i4 usually used in routine applications. However, despite the higher overall image quality score, the very pronounced image smoothing using the Smoother level was not retained for routine clinical use. Based on our preliminary phantom study [16] and on a patient study for abdominal CT examinations [30], the Smooth level was chosen for parenchymal images in oncology patient follow-up. The Smoother level could be used in Ultra-low dose chest CT acquisitions or ultra-low dose chest-abdomen-pelvis CT acquisitions where a high noise level in the images limits the impact of image smoothing.

The limitations of this study are as follows: first, only a small number of patients from a single institution were included and only one CT system was used. Second, those included were not enrolled for chest CT acquisitions alone, but for chest-abdomen-pelvis acquisitions performed as part of the oncological follow-up of patients with known lung lesions. Different outcomes may be found with other chest lesions. Furthermore, this study only assessed the subjective image quality and the accuracy of diagnosis using size measurements, not the number of lesions. It would be interesting to confirm the potential of this deep-learning image reconstruction algorithm via a prospective study with a larger patient population. Also, only one dose level was used whereas this algorithm has a potential for dose reduction that was not assessed by this study. The generalizability of our study results must now be confirmed by further patient studies to assess this new algorithm’s potential for detecting solid lung lesions via ultra-low dose CT acquisitions.

## 5 Conclusion

In conclusion, according to our study, the Smooth and Smoother levels of the new deep-learning image reconstruction algorithm reduced the image noise and improved the contrast-to-noise ratio and overall and diagnostic image quality without changing the chest tumor size compared to iterative reconstruction algorithms. The Smoother level of this new deep-learning image reconstruction algorithm gave the highest scores for the overall and the diagnostic quality of the chest images. This new algorithm may now be used in clinical practice for chest CT acquisitions in chest tumor follow-up.

## Ethical statement

The study was conducted in accordance with the Declaration of Helsinki (as revised in 2013) and approved by the institutional review board of the Nîmes University Hospital. Participants and/or their legal guardians were systematically informed that details were being collected for an anonymous retrospective study and could refuse to participate in it at any time.

## Author’s contribution

(I) Conception and design: JG, JF

(II) Administrative support: JPB

(III) Provision of study materials or patients: JG, JF and JBP

(IV) Collection and assembly of data: JG, DD, JF

(V) Data analysis and interpretation: JG, JF, MP, QD, RS, CS

(VI) Manuscript writing: All authors

(VII) Final approval of manuscript: All authors

## Declaration of competing interest

The authors declare that they have no known competing financial interests or personal relationships that could have appeared to influence the work reported in this paper.
